# Pushing the boundaries of digital social contact: Experiences of people with disabilities and their social networks during the COVID-19 pandemic

**DOI:** 10.1177/17446295231210021

**Published:** 2023-10-25

**Authors:** Lianne Bakkum, Lotte Piekema, Linda Douma, Carlo Schuengel, Paula Sterkenburg, Esmee Adam, Annet ten Brug, Noud Frielink, Petri Embregts, Anne Tharner

**Affiliations:** Department of Educational and Family Sciences, Amsterdam Public Health Research Institute, 1190 Vrije Universiteit Amsterdam, Amsterdam, the Netherlands; Viveon; Department of Inclusive and Special Needs Education and Child Care, University of Groningen, Groningen, the Netherlands; Academische Werkplaats EMB; Department of Educational and Family Sciences, Amsterdam Public Health Research Institute, 1190 Vrije Universiteit Amsterdam, Amsterdam, the Netherlands; Viveon; Department of Educational and Family Sciences, Amsterdam Public Health Research Institute, 1190 Vrije Universiteit Amsterdam, Amsterdam, the Netherlands; Academische Werkplaats Sociale relaties en gehechtheid; Academische Werkplaats Leven met een verstandelijke beperking, Tranzo, Tilburg School of Social and Behavioral Sciences, 120694 Tilburg University, Tilburg, the Netherlands; Department of Public Health and Primary Care, Leiden University Medical Center, the Netherlands; Department of Inclusive and Special Needs Education and Child Care, University of Groningen, Groningen, the Netherlands; Academische Werkplaats EMB; Academische Werkplaats Leven met een verstandelijke beperking, Tranzo, Tilburg School of Social and Behavioral Sciences, 120694 Tilburg University, Tilburg, the Netherlands; Department of Educational and Family Sciences, Amsterdam Public Health Research Institute, 1190 Vrije Universiteit Amsterdam, Amsterdam, the Netherlands; Viveon

**Keywords:** COVID-19, information and communication technology, informal social networks, social contact, well-being

## Abstract

During the COVID-19 pandemic, many people with intellectual disabilities living in care facilities could not receive visitors. Health authorities suggested the use of digital social contact as an alternative for in-person visits. We examined how people with intellectual disabilities living in care facilities experienced the use of digital social contact with their informal social network throughout 2020. Residents, their relatives, volunteer visitors, direct support staff, and care facility managers (*N* = 283) completed an online questionnaire, of whom 35 participated in an interview. Video calling and in-person visits were among the most common forms of staying in touch. Experiences with digital social contact depended on residents’ abilities and support needs, and on preconditions, such as staff availability. The first phases of the pandemic led to experiences of possibilities and benefits of using digital social contact as complementary to in-person contact for people with different levels of intellectual disability, also after the pandemic.

## Introduction

During the COVID-19 pandemic, national governments worldwide imposed restrictions on in-person visits to people living in long-term care facilities (Salcher-Konrad et al., 2020). In the Netherlands, the government introduced the first lockdown period from March until May 2020. Sheltered care facility homes adopted a policy under which no visitors or outside visits were allowed, except for urgent or compassionate situations (Vereniging Gehandicaptenzorg Nederland, 2020). This policy was eased after May 2020, although some restrictions were kept in place. Health authorities (e.g., World Health Organization, 2020) suggested the use of digital technologies, such as video calling, as an alternative for in-person visits. This study investigated how people with intellectual disabilities living in sheltered care facility homes and their informal social networks stayed in touch throughout 2020. In addition, we examined how they experienced the use of digital social contact during this period.

Informal social networks (e.g., family and friends) are important for providing support to people with intellectual and multiple disabilities (Kamstra et al., 2015; van Asselt-Goverts et al., 2013). Additionally, contact with family and friends is imperative for meeting their basic psychological needs of relatedness and autonomy (Ryan and Deci, 2000; Frielink et al., 2018). Therefore, the pandemic restrictions may have negatively impacted the needs and daily lives of people with disabilities. They missed having in-person contact with family and friends during the pandemic lockdown (Embregts et al., 2020; Honingh et al., 2021) and some felt ‘locked-up’ within their sheltered care facility home (Honingh et al., 2021).

Digital social contact may help people with disabilities to stay in touch with their informal social networks, though they may experience more difficulties with using digital technology than people without disabilities (Chadwick et al., 2013) and may have less access to the digital world (Alfredsson Ågren et al., 2020). Support in using digital social contact may, therefore, be an important condition (Navas et al., 2020). Previous studies have addressed what means of digital social contact are used by people with intellectual disabilities (e.g., Danker et al., 2023; Lancioni et al., 2020; Ramsten et al., 2020), potential effects on their well-being (e.g., Chadwick et al., 2023; Shpigelman & Gill, 2014), and the risks and benefits of the use of digital social contact (e.g., Chadwick et al., 2016, 2023). However, insight into the use of digital social contact by people with disabilities living in sheltered care facility homes is limited (Bakkum et al., 2022), with few relevant studies conducted during the pandemic (e.g., Araten-Bergman & Shpigelman, 2021; Caton et al., 2022; Chadwick et al., 2023; Spassiani et al., 2022). For example, Araten-Bergman and Shpigelman (2021) explored how family caregivers of people with intellectual disabilities stayed in contact with their relatives living in care facilities during the first lockdown period in Israel. Although most families used digital social contact to stay in touch, family caregivers reported having limited opportunities for providing social support. A limitation of this study, noted by the authors themselves, is that they only relied on family caregiver self-reports. Chadwick and colleagues (2023) interviewed people with intellectual disabilities about the role of ICT in their everyday lives during the pandemic, and provided insight in facilitating factors and barriers of ICT use and the impact of digital social contact on their well-being. However, this study could only provide limited insight into the nature and types of support with using ICT, because the perspectives of those providing support were not included. To better understand the experiences and needs of people with intellectual disabilities, it is important to include their own perspectives, as well as the perspectives of people from their informal and formal social network (Araten-Bergman et al., 2021; Chadwick et al., 2023). This may not only be important for future situations in which in-person contact is temporarily restricted, but also for enabling their inclusion and participation in the digital world (Chadwick et al., 2022).

The current study adds to previous work by exploring the experiences and perspectives of people with intellectual disabilities using digital social contact during the COVID-19 pandemic, as well as those of their family, volunteer visitors, direct support staff, and care facility managers. Using complementary quantitative and qualitative research methods, we addressed the following research questions:

(1) How did people with disabilities stay in contact with their relatives and volunteer visitors throughout 2020 while COVID-19 visiting restrictions were in place, and how did people with disabilities, their relatives, volunteer visitors, direct support staff and care facility managers experience these ways of staying in contact?
(2) How did the use of digital social contact during this period impact the well-being of people with disabilities, and how did their relatives, volunteer visitors, direct support staff, and care facility managers perceive this potential impact?
(3) What were the support needs and conditions for the use of digital social contact during this period according to people with disabilities, their relatives, volunteer visitors, direct support staff, and care facility managers?

## Material and methods

### Study design

A multi-method, cross-sectional design was used, consisting of online questionnaires and semi-structured interviews. Anonymous questionnaires were sent out to five participant groups: (1) people with mild/moderate disabilities living in sheltered care facility homes (residents), (2) relatives of residents, (3) volunteer visitors, (4) direct support staff, and (5) care facility managers. Five different questionnaires with overlapping content were created. After filling in the questionnaire, participants were asked if they were willing to participate in an interview. Interview protocols were created for each participant group, guided by topics from the online questionnaire. The interviews were held via video calling. The questionnaires and interviews were analysed separately. The study protocol is available on Open Science Framework: https://osf.io/pkbmq

### Participants

This study was a collaboration between three Dutch universities, four Academic Collaborative Centres and client advocacy bodies for people with disabilities. Participants were recruited through these organisations and through online platforms such as the project’s website, social media outlets, and digital newsletters sent out to health care organisations. In total, 283 participants responded to the online questionnaires. Participants were 31 residents, 80 relatives of residents, 72 volunteer visitors, 82 direct support staff, and 18 care facility managers.

Residents were eligible for inclusion if they lived in a 24-hours sheltered care facility home for people with intellectual or multiple disabilities and if they were able to fill in the questionnaire independently or with support from a family member or direct support staff, meaning that these residents had a mild-to-moderate intellectual disability. Relatives and volunteer visitors were included if they had a relative or worked as a volunteer visitor for someone with a disability (mild, moderate, severe or multiple) who lived in a 24-hours sheltered care facility home. Direct support staff and care facility managers were included if they worked at a 24-hours sheltered care facility home for people with disabilities. All participants had to be 16 years or older to be included. Demographics of the final sample are reported in Table 1.

**Table 1.** Demographics of Questionnaire Participants (N = 283).

	Residents	Relatives	Volunteer visitors	Direct support staff	Care facility managers
	*n* (%)	*n* (%)	*n* (%)	*n* (%)	*n* (%)
Subsample size	31	80	72	82	18
Gender					
Female	15 (48)	63 (79)	44 (61)	71 (87)	15 (83)
Age					
18-24	5 (16)	1 (1)	2 (3)	7 (9)	--
25-64	25 (81)	51 (64)	34 (47)	73 (89)	18 (100)
> 65	1 (3)	28 (35)	36 (50)	2 (2)	--
Living situation of residents					
With others, with support	20 (65)	75 (94)	57 (79)	76 (93)	16 (89)
Independently, with support	11 (35)	6 (6)	15 (21)	6 (7)	2 (11)

*Note*. Questionnaire participants were not asked about the level of intellectual disability.

Considering both expected data saturation and time constraints, we initially aimed to include eight to ten participants per target group in the interviews. Seventy questionnaire respondents were willing to be interviewed, of whom 58 were invited to take part. This included all residents, volunteer visitors, direct support staff, and care facility managers who expressed an interest to be interviewed. Residents were invited for an interview if they could verbally express their opinions about digital social contact. Many relatives wanted to be interviewed, therefore, not all of them were invited to take part. Of the 58 people who were invited, 35 participated: 5 residents, 11 relatives, 9 volunteer visitors, 6 direct support staff members, and 4 care facility managers. Reasons for not participating were lack of response or difficulties with scheduling the interview.

### Procedure

Recruitment took place between 15 January and 2 April 2021. After reading the information, participants were guided to the project's website to sign up for the online questionnaire. Anonymous questionnaires were sent out using Survalyzer. To secure anonymous data collection, questionnaire participants were redirected to a separate online form if they wanted to participate in an interview. The interviews were held via video calling (Google Meet, Skype or Microsoft Teams) in March and April 2021, by three researchers. One researcher conducted the interviews with residents and direct support staff, one conducted the interviews with relatives, and another researcher conducted the interviews with volunteer visitors and care facility managers. Residents could have a support staff or family member present during the interview. The interviews lasted 30-60 minutes, depending on how much the interviewee had to share. Recordings were made via video calling software and transcribed verbatim.

Participants gave written informed consent for taking part in the questionnaires and interviews. Where applicable, written informed consent was given by legal representatives as well. The collection of personal data was limited to the names and e-mail addresses of the participants who wished to participate in an interview. The study protocol was approved by the Scientific and Ethical Review Board of the Faculty of Behavioural and Movement Sciences of Vrije Universiteit Amsterdam (VCWE-2020-185) and the Ethical Committee Pedagogical and Educational Sciences of Rijksuniversiteit Groningen (9-12-2020).

### Measures

Questionnaires and interview protocols were developed by the research team and included feedback and review from a panel of experts-by-experience (people with mild/moderate disabilities and relatives). In both the questionnaires and interviews, residents, their relatives and volunteer visitors were instructed to report about their own experiences during 2020. For the questionnaire, direct support staff were asked to keep in mind the resident with the highest care needs they supported. Care facility managers were instructed to answer the questions about the residents they were most involved with. In the interviews, direct support staff and care facility managers were asked about all resident groups they were involved with, and to describe potential differences between these groups.

#### Questionnaires about digital social contact

The questionnaires contained closed-ended and open-ended questions. The content of the questionnaires and the number of items differed slightly for each group (27 items for residents, 32 items for relatives, 31 items for volunteer visitors, 36 items for direct support staff, and 18 items for care facility managers). All participants were asked to report on their demographic characteristics, such as gender and age-category, and direct support staff and care facility managers reported on their education and work experience. Residents, relatives, volunteer visitors, and direct support staff were asked with whom the residents had contact with and in what way (e.g., in-person visits, window visits, digital social contact) throughout 2020, how they evaluated these ways of social contact, how the use of digital social contact had impacted the well-being of residents, and how residents experienced and perceived support needs and conditions for the use of digital social contact. These participant groups were also asked about the living situation of residents (whether they lived on their own or with others, and how far away from relatives they lived), the difficulties that residents experienced (e.g., cognitive or physical skills, visual/hearing impairments, behavioural problems), and to what extent they thought these difficulties impacted the use of digital social contact. Relatives, volunteer visitors, direct support staff, and care facility managers answered additional questions about their view on practical preconditions for digital social contact.

#### Interviews about digital social contact

The semi-structured interview protocols were guided by topics from the online questionnaire. The interviews focused on topics that were difficult to ask in-depth about in a questionnaire, such as reasons for using or not using digital social contact and residents’ difficulties with using digital social contact. The interview protocols for direct support staff and care facility managers also included questions about the organisation’s policy on the use of digital social contact, before and after the pandemic. In addition, participants were asked background questions about themselves (e.g., education and work experience), the resident (i.e., disability and severity of the disability), and the sheltered care facility home (e.g., residents living on their own or sharing an apartment with others).

### Data analysis

The questionnaires and the interviews were analysed separately for answering the research questions. R version 4.0.5 (R Core Team, 2021) and the *tidyverse* package (Wickham et al., 2019) were used to analyse the questionnaire responses. Frequency and descriptive statistics were calculated, including confidence intervals where appropriate.

The interviews were analysed with Atlas.ti Version 8.0 (Atlas.ti, Scientific Software Development GmbH, 2020) using thematic analysis (Braun & Clarke, 2006) and following the phases of open, axial, and selective coding. The interviews were independently coded by the three researchers who conducted the interviews. Each researcher analysed their own participant group: one researcher coded the interviews of residents and direct support staff, one researcher coded the interviews of relatives, and one researcher coded the interviews of volunteer visitors and care facility managers. To establish inter-rater reliability, the three researchers coded the first interview of a relative independently, after which the codes and group themes were discussed until consensus was reached. A concise list of codes and group themes was then developed. Next, the researchers each coded two interviews of their own respondent groups. Codes and group themes were modified when necessary through discussion. After this, the remaining interviews were coded. Weekly meetings were held to discuss the coding process. Connections between themes were identified and final themes were discussed during regular meetings with all authors of this article.

## Results

### Descriptive statistics

All questionnaire participants reported on residents’ difficulties with various cognitive or physical skills, visual/hearing impairments, and behavioural problems, as well as on whether these difficulties impacted the possibilities of using digital social contact. These findings are reported in Table 2.

**Table 2.** Residents’ Difficulties With Cognitive and Physical Skills, Visual and Hearing Impairments, and Behavioural Problems.

	*n*	%
Reading or writing	191	68
Thoughts or feelings	191	68
Planning	189	68
Learning	183	66
Self-care	179	64
Activities of daily living	160	57
Behaviour	150	54
Talking	134	48
Memory	126	45
Walking	121	43
Using hands	96	34
Vision	57	20
Other difficulties	42	15
Hearing	38	14
Do the resident's difficulties impact their ability to use digital social contact?		
No	117	42
Yes, a little bit	78	28
Yes, very much	66	24

*Note*. Residents’ difficulties were reported by all participant groups. (i.e., residents, relatives of residents, volunteer visitors, direct support staff, and care facility managers).

### Ways of staying in contact throughout 2020, and experiences with digital social contact

#### Ways of staying in contact

**Figure 1.**
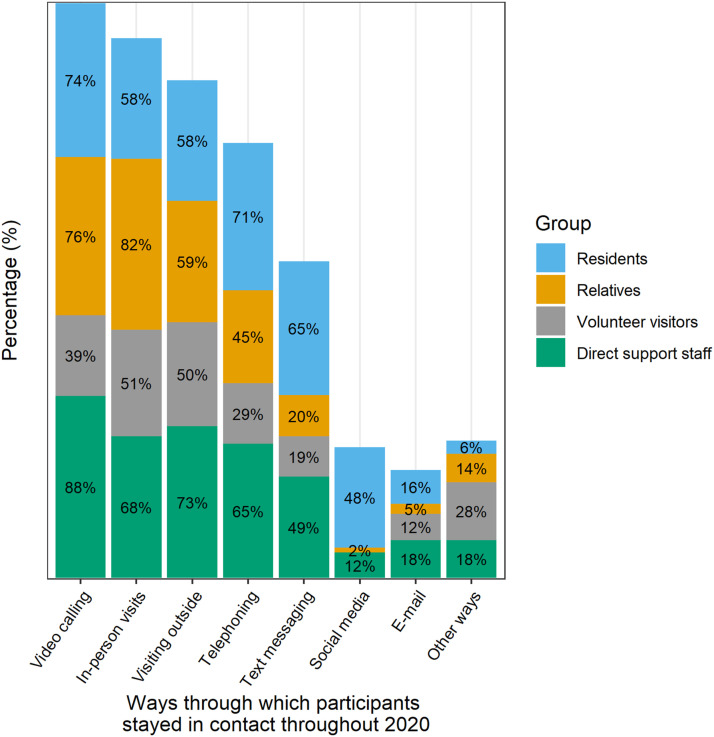
The ways through which residents stayed in contact with their informal social networks throughout 2020. It was possible to give more than one answer.

The ways through which residents stayed in contact with their informal social networks throughout 2020, as reported in the questionnaires, are reported in Figure 1. Video calling, in-person visits, and outside visits were the most commonly reported ways of staying in touch. It is important to note that the initial strict visiting restrictions were eased two months after the government announcement of the first lockdown, making it possible for relatives and volunteer visitors to visit again, although still under restrictions.

The results of the interviews showed that most residents with mild-or-moderate disabilities had already used some form of digital social contact before the pandemic and started using it more often during the pandemic. According to direct support staff, most residents with severe or multiple disabilities had not used digital social contact before the pandemic, but started using it to stay in touch after the lockdown restrictions were imposed. Direct support staff facilitated the use of digital social contact, such as video calling and livestreams during special occasions (e.g., birthdays or holidays). Digital social contact with relatives, friends, or volunteer visitors were more challenging for residents with difficulties speaking and residents who depended on hand gestures and touch for communication. For some residents, pictures, videos, and text messages sent by direct support staff to their families were the only forms of digital social contact they had with their families.

Digital social contact was used in more ways than having conversations. Relatives, volunteer visitors, and direct support staff indicated that digital social contact was also used for alternative activities, such as wishing goodnight, singing songs, or playing games, as illustrated by this quote:

*“At that moment we arranged a bingo event with the girls over there. It actually worked using video calling.”* [Volunteer visitor 4]

In addition, video calling was sometimes used to provide practical support:

*“Video calling can be such an advantage. Sometimes our son receives letters from the government, such as his voting pass, or he gets a postcard from someone. Support staff are not always able to read his mail for him, or he does not understand it. Or he just wants to show it to me. He then puts it in front of the camera so that I can read it for him.”* [Relative 2]

As reported in the questionnaires, most residents (77%), relatives (80%), and direct support staff (76%) preferred having both in-person contact and other ways of contact (e.g., digital communication). This was preferred by 43% of volunteer visitors (not reported in a Figure).

In the interviews, volunteer visitors reported using little digital social contact. Volunteer visitors explained that this might have to do with reasons why they became a volunteer visitor in the first place: doing fun activities together in-person and making sure the resident has a good time. Volunteer visitors started getting back to in-person contact as soon as they were allowed to visit again.

Relatives, volunteer visitors, direct support staff, and care facility managers reported that digital social contact was more frequently used than in-person contact during the restrictions, and many would continue using digital communication when visiting restrictions are to be lifted. However, they also noted that digital social contact could not compensate for the inherent value of in-person contact. It could provide a temporary alternative to in-person visits, as indicated by a direct support staff member:

*“Of course, we stayed in touch through FaceTime and Skype. We felt like the loss of not seeing her parents in-person was not really severe, though I think this is because we called her parents every day so she could hear their voices.”* [Direct support staff 1]

One volunteer visitor used digital social contact to compensate for activities they used to do in-person:

*“I recorded a few songs for one of the children. Support staff played these for him occasionally. He enjoyed listening to these songs, in the same way as he enjoys listening to other music. … It has been quite a challenge to find out how to compensate for something that you cannot do in-person.”* [Volunteer visitor 2]

Residents, relatives, and direct support staff used video calling more often than telephoning without video, because of the advantage of seeing one another and getting a better sense of how the other person is doing. Some residents preferred video calling over telephoning or texting when they had much to talk about. An additional advantage of using video calling was that they did not need phone credit for video conversations because they could use the organisation’s WiFi network.

#### Experiences with using digital social contact

**Figure 2.**
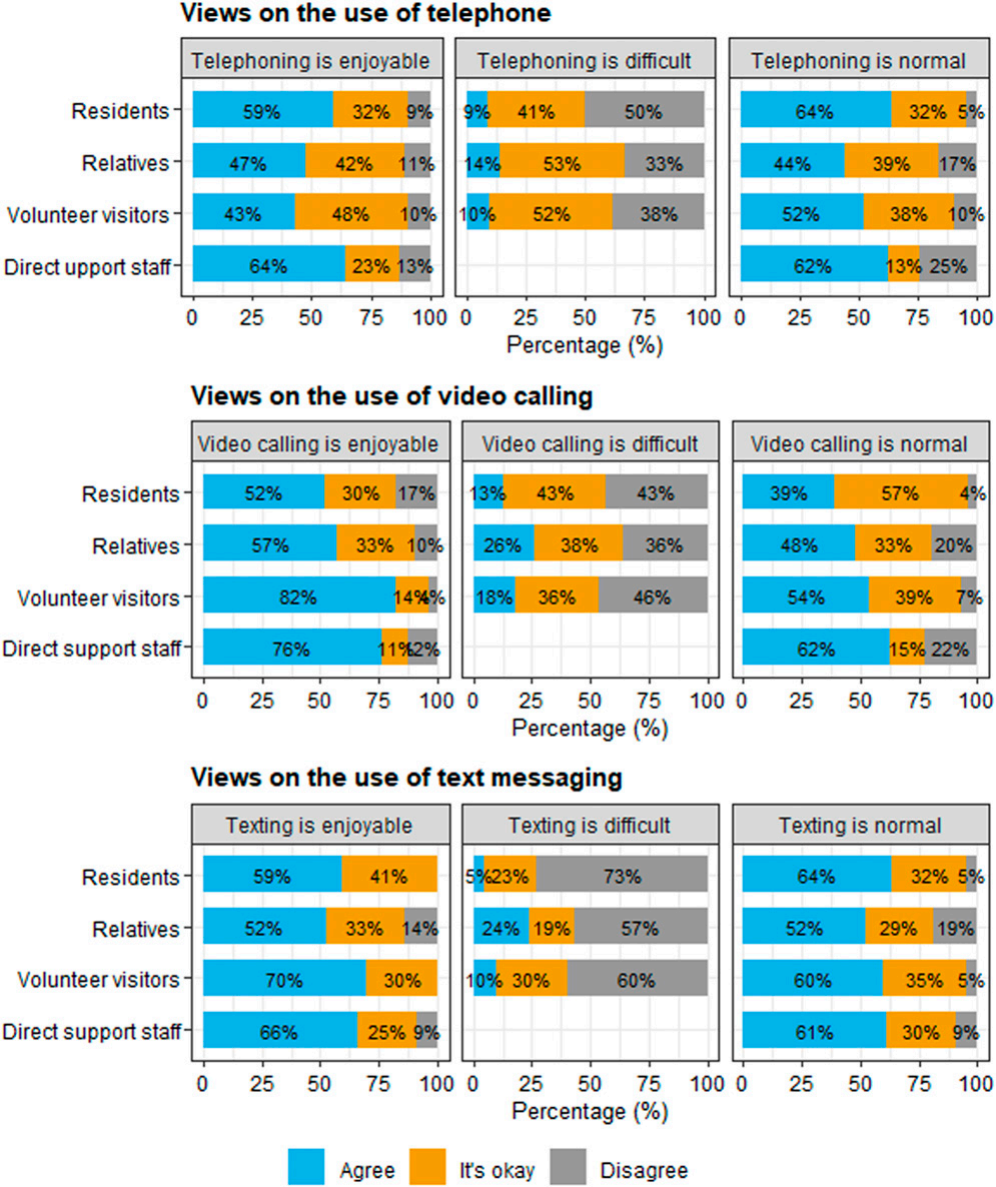
Participants’ views on using the telephone, video calling, and text messaging. Percentages are rounded and therefore do not always sum to 100%.

In the questionnaires, participants reported on their experiences with telephoning, video calling, and text messaging in terms of how enjoyable, difficult, and normal they found these ways of digital social contact. These findings are reported in Figure 2. Participants seemed to agree that using the telephone, video calling, and sending text messages are enjoyable or ‘okay’. However, there was little agreement between participants about how difficult or normal these forms of communication are.

In the interviews, participants elaborated on their experiences with digital social contact. All participants considered in-person contact to be important. Nonetheless, most recognised the benefits digital social contact could bring them during visiting restrictions. For example, relatives and volunteer visitors noted benefits of video calling, such as seeing how residents were doing. However, some residents mentioned that digital social contact lacked physical contact, as illustrated by the following quote:

*“Yeah, it is a little bit boring, but we must deal with it. It is good that there are other possibilities, but I do not enjoy these so much, because I miss having physical contact.”* [Resident 2]

Residents’ difficulties with using digital social contact depended on the level of disability, their digital skills, and prior experience with digital social contact. Some residents were already familiar with some kind of digital social contact (e.g., text messaging or video calling), which made it easier to learn another kind of digital social contact. Residents with visual or hearing difficulties relied mainly on in-person contact. Nonetheless, some residents with a visual impairment did engage in video calling, mainly because it allowed their relatives to see them.

Some difficulties with video calling were practical in nature, such as the small screen size:

*“At first he found it really interesting and fun to call with mum and dad. But he uses a lot of hand gestures, and these are not always visible on a small screen. … You want to see your child, but the screen is actually too small to see both their face and their hands.”* [Relative 6]

Other difficulties were cognitive in nature, such that some residents found it difficult to understand the concept of video calling:

*“At one point during the pandemic, one of the residents was very sad. I asked him: ‘Shall we call your brother?’. During the three weeks after the call, the resident kept asking if his brother was still in my phone. He really thought his brother was in my pocket. They do not always understand how it works. Using a phone is difficult sometimes, but video calling…. They can see someone, but they cannot touch them. Video calling is not for everyone.”* [Care facility manager 1]

For some residents, the intensity and attention span needed for video conversations were challenging. One resident stated that video calling with more than one person was draining because of the need to process more stimuli. Relatives, volunteer visitors, and direct support staff noted that some residents were easily distracted during video calls. For example, residents seemed to often look away from the screen and the conversations were mostly short. As a solution, one relative walked around the house in the way they would normally do when the resident came to visit.

Difficulties with reading or writing hindered the use of text messaging, as reported by residents, relatives, and volunteer visitors. One volunteer visitor noted that a resident was able to read and write text messages, but that it was difficult to understand the context. Some residents needed support with composing text messages, as illustrated by the following quote:

*“I find writing quite difficult, because I cannot write very well. Some words are quite difficult to type and then I need to ask for support.”* [Resident 4]

When asked in the questionnaires to report their satisfaction with digital social contact on a scale of 1-10 (1 = *not satisfied at all*, 10 = *completely satisfied*), residents scored on average 7.43 (*SD* = 2.21, 95% CI [6.62, 8.24]), relatives scored on average 7.46 (*SD* = 1.46, 95% CI [7.14, 7.79]), and volunteer visitors scored on average 7.02 (*SD* = 1.94, 95% CI [6.57, 7.48]). Direct support staff scored on average 7.33 (*SD* = 1.48, 95% CI [7.01, 7.66]) for satisfaction of digital social contact between the resident and their informal social networks. These scores suggest that participants seemed satisfied with the use of digital social contact.

### The impact of using digital social contact on the well-being of residents

**Figure 3.**
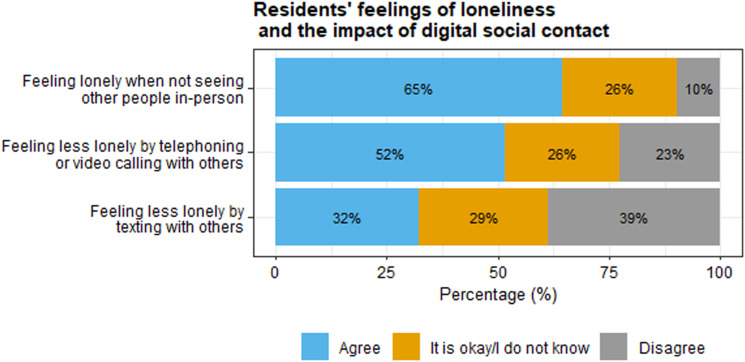
Feelings of loneliness and the impact of digital social contact, as reported by residents. Percentages are rounded and therefore do not always sum to 100%.

In the questionnaires, residents reported on the impact of using digital social contact on feelings of loneliness. As seen in Figure 3, many residents reported feeling lonely when not seeing other people in-person, and there was little agreement about the extent to which video calling and texting made them feel less lonely.

Interview responses indicated that most residents experienced life to be less structured during the lockdown restrictions than before. This led to agitation in some residents, while others felt more relaxed. Relatives, direct support staff, and care facility managers in some residents observed more behavioural problems (e.g., aggression) during the restrictions. Some direct support staff noticed that residents seemed lonelier than before. For some residents, digital social contact with loved ones could mitigate the impact of the restrictions on their well-being, such that it made them feel less lonely. These residents appreciated the digital conversations with others. This was also noticed by direct support staff, who mentioned that some residents seemed happy, energised, and talkative during video calls with relatives. Most relatives also indicated positive digital interactions with their family members. However, several relatives and direct support staff noticed that some residents seemed sad during video calls, because it reminded them that in-person contact was not possible. One care facility manager mentioned that some residents seemed happier with a postcard than a video conversation.

The impact of using video calling on the well-being of residents with severe or multiple disabilities was less clear compared to residents with mild-to-moderate disabilities. According to some relatives, these residents might have trouble understanding what is happening or with recognising the other person on a screen. Nonetheless, they may still enjoy or value the personal interaction:

*“We say goodnight when he goes to bed. He sings a song for us or makes some sounds. We try to tell him when we will see each other again. We are not completely sure what he understands of our interaction, but we think that he is able to understand part of it. And he enjoys it, we can see how he responds to us. So this is actually something we can continue doing.”* [Relative 10]

The lockdown restrictions also had an impact on relatives of residents, especially during the first phase when no in-person visits were allowed. Direct support staff noticed that parents found it difficult to not see their child in-person for a prolonged time. Some parents of a child with severe or multiple disabilities were afraid that their child would not recognise them anymore after not seeing them for so long. In addition, some relatives felt denied in their role as a parent or caregiver as decisions about restricting social contact were made without them. Due to the loss of in-person contact, some parents were confronted with the realisation that their child was not part of their household anymore:

*“I could visit her at one point and that was nice, but I could not take her home with me. So she misses out on a lot of our family activities. In fact, she does not really belong to the family anymore, if you know what I mean. She has her own life, and we do many things together as a family, so she misses out on a lot.”* [Relative 4]

### Support needs and conditions for digital social contact

#### Practical conditions and supplies

**Figure 4.**
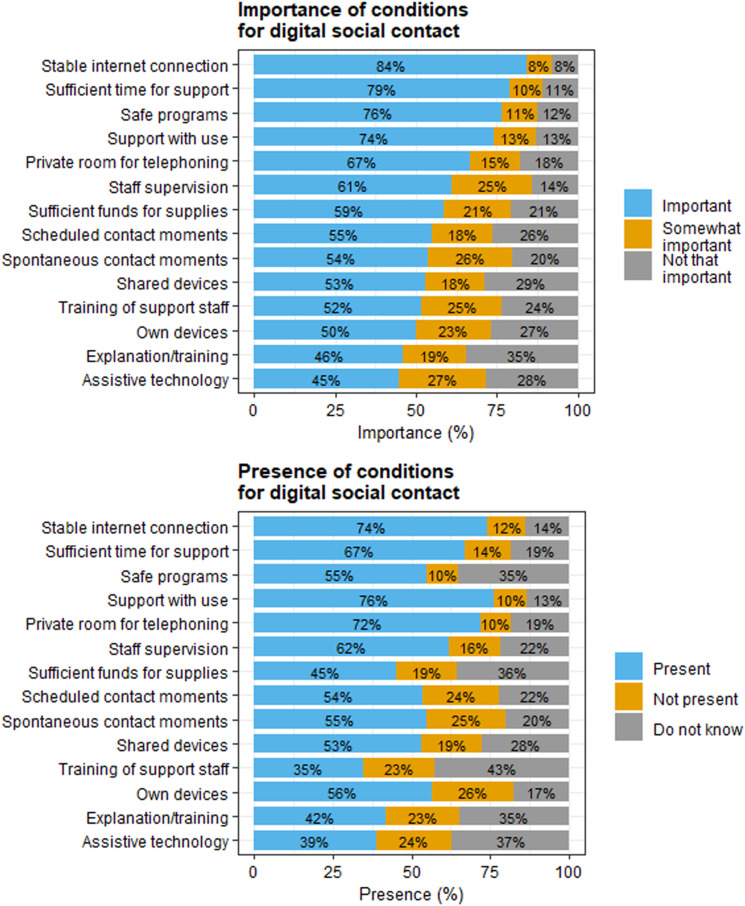
Importance and presence of conditions for digital social contact, according to relatives, volunteer visitors, direct support staff, and care facility managers. Note: care facility managers reported on the presence but not on the importance of conditions. Percentages are rounded and therefore do not always sum to 100%.

Questionnaire participants reported on the importance and presence of conditions for digital social contact. These conditions included both contextual conditions (i.e., ways in which the sheltered care facility home could meet the needs of residents and relatives) and supplies for facilitating digital contact, such as devices. Figure 4 presents the importance and presence of conditions for digital social contact, according to relatives, volunteer visitors, direct support staff, and care facility managers. The three *most important* conditions for digital social contact were a stable internet connection, sufficient time for support, and safe programs. The top-three conditions that were *present* in sheltered care facility homes were support with use of digital social contact, a stable internet connection, and a private room for telephoning.

In the interviews, residents with mild-to-moderate disabilities mostly used their own devices for having digital social contact, whereas residents with severe or multiple disabilities mostly did not own devices. In the beginning of the lockdown restrictions, some direct support staff used their personal devices for facilitating contact between residents and relatives. These were later replaced by facility-owned devices, partly due to privacy concerns. Although relatives, volunteer visitors, direct support staff, and care facility managers considered the facilitation of digital social contact a responsibility of care organisations, they differed in their views on who is financially responsible. Some concluded it was the responsibility of residents and their family, while others concluded it was the responsibility of the sheltered care facility homes, or both.

Digital conversations between residents and their relatives or visiting volunteers were sometimes held in a common room, such as the living room, and sometimes in the residents' own apartment or a private office. Relatives, volunteer visitors, and direct support staff noticed that digital conversations were less open and intimate with other people around than when conducted in private, and that the presence of others made residents more distracted during conversations:

*“A quiet environment allows for them to focus on the conversation, because they are easily distracted. With one resident, we tried to have a video conversation at the kitchen table, but the room was crowded with other*s*, which distracted him from the conversation. Now we use a private office so he can focus on the conversation and does not get distracted. And that is important I think, especially for the residents I work with.”* [Direct support staff 2]

Some factors hindered the facilitation of digital social contact. Volunteer visitors mentioned that direct support staff working in shifts made it difficult to organise digital social contact. Problems with arranging digital contact moments were solved by scheduling appointments with residents and direct support staff. Another hindering factor, mentioned by some residents, relatives, and direct support staff, was an unstable internet connection.

#### Support needs

**Figure 5.**
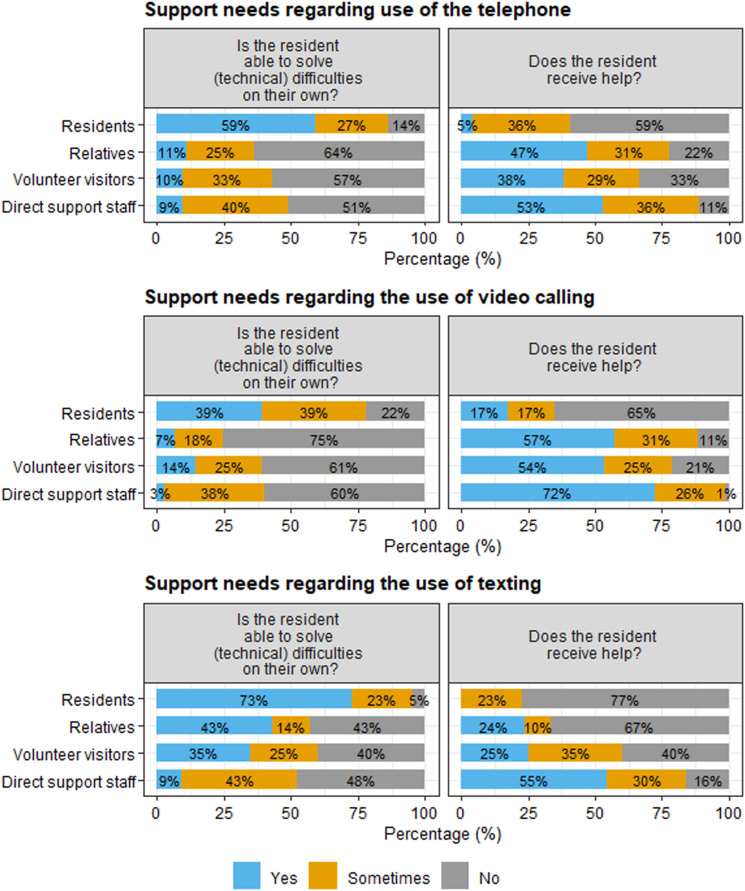
Residents’ abilities to solve problems with digital social contact and (perceived) support needs, according to residents, relatives, volunteer visitors, and direct support staff. Percentages are rounded and therefore do not always sum to 100%.

Questionnaire participants were asked to report on residents’ support needs with regard to digital social contact. As seen in Figure 5, participants differed in their perceptions on residents’ abilities to solve problems with digital social contact on their own, and on the extent to which they receive support with the use of digital social contact. It is important to note that residents with severe or multiple disabilities did not participate in the questionnaire, which may partly explain these different perspectives.

In the interviews, the extent to which residents needed support with using digital social contact depended on their reading and writing skills and their intellectual and physical abilities. Some residents with mild-to-moderate disabilities were able to initiate and use digital social contact independently. Direct support staff noticed that some residents with a moderate intellectual disability needed support with technical issues. Other residents did not need technical assistance from direct support staff, but needed help with staying focused on the conversation and with translating non-verbal signals during video calling. Other ways in which direct support staff and relatives provided support included explanation and training with new applications. Some care organisations offered courses for residents to learn how to use devices and applications. Residents with severe or multiple disabilities needed both technical support and support with keeping the conversation going. While some residents appreciated the help from direct support staff, others found it annoying because it made them feel less independent:

*“Sometimes I find it annoying to need help from others. Sometimes I think: ‘I am going to send a text message on my own’ and see how the other person responds. […] I like to be independent, you know, and support staff do not always have time to help. […] There are other residents as well, so I do not always want to ask for help.”* [Resident 4]

Not all direct support staff were able to provide support. Older staff reported more difficulties with digital social contact than younger staff, which older staff solved by asking support from younger staff. In some organisations, courses for digital social contact were available to direct support staff, but their high workload did not always allow them to take these courses. Some support staff members expressed concerns regarding the potential risks of digital social contact, especially the use of texting and social media, as illustrated by this quote:

*“We have come across a situation in which a young male resident with a mild intellectual disability texted a picture of his genitals to a female resident of another care facility home. Well, how are you going to deal with that?”* [Care facility manager 2]

Staff shortages and the amount of day-to-day tasks were reasons why direct support staff were not always able to provide help. Direct support staff tried to overcome this by scheduling time for digital social contact during shifts with sufficient staff. Overall, in the interviews participants noticed that the amount of support with digital social contact varied throughout the different periods of COVID-19 restrictions. Volunteer visitors noticed that direct support staff actively thought about digital social contact as part of daily care in the first phase of the pandemic, but that this attention declined in later stages of the pandemic. Also relatives noticed that they were less involved in the organisation of digital social contact with the resident in later stages of the pandemic.

## Discussion

During the COVID-19 pandemic in 2020, residents, relatives, and volunteer visitors mostly stayed in contact with each other by in-person visits and video calling. Telephoning and text messaging were less commonly used. Participants described benefits of digital social contact, such as an easy and accessible way to stay in touch between in-person visits, but they also reported difficulties and disadvantages. Residents’ difficulties with digital social contact were both practical (e.g., problems with typing) and cognitive in nature (e.g., not understanding what is happening during video calling). Digital social contact made some residents feel less lonely during the pandemic, while it caused confusion or sadness in others. Furthermore, participants provided insights into the importance and presence of conditions of digital social contact, both in terms of practical conditions and supplies and in terms of support needs of residents.

### Experiences with digital social contact

In this study, video calling was commonly used by residents to stay in contact with their informal social networks, which is consistent with previous studies conducted during the pandemic (e.g., Araten-Bergman & Shpigelman, 2021; Chadwick et al., 2023; Embregts et al., 2020; Honingh et al., 2021). Participants' experiences with the use of video calling were mixed. Our findings indicate that video calling can be possible and enjoyable, even for people who were not able to communicate verbally. Video calling was used for having conversations, but also for other activities, such as a night-time ritual or singing songs together, or for observing how someone is doing. When participants were allowed to visit again, video calling was continued to be used as a complementary form of contact. For example, digital social contact made it easier to communicate between in-person visits, when someone was ill or on holiday, or when a person lived further away from the sheltered care facility home.

Whether an individual used digital social contact depended on multiple factors, such as their personal interests and abilities, the medium used, the possibility to practise, and the presence of a supporting environment. Although training and support with use were perceived as important conditions for digital social contact, there may not have been sufficient time to practise and support during the early stages of the pandemic. Most residents with mild-to-moderate intellectual disability in our study already used digital social contact before the pandemic, and continued using it during the lockdown, which is consistent with previous findings (Chadwick et al., 2023). Although video calling may be more challenging for those with severe/profound or multiple disabilities, our study showed this was valuable and enjoyable for some residents, while causing distress in others. This adds to previous findings by Caton et al. (2023), who reported on the experiences of family members and support workers of people with profound and multiple intellectual disabilities. As these authors noted, for some individuals, new experiences with digital social contact may further develop after the pandemic. Further work is needed to examine how people with severe/profound and multiple disabilities may be supported using digital forms of contact, such as video calling.

### Well-being

The loss of in-person contact with family and friends made some residents in our study feel lonely. For some, digital social contact could mitigate their feelings of loneliness. For others, digital social contact emphasised the lack of in-person contact. Mixed results about digital social contact and loneliness were also found by Caton et al. (2022), who surveyed more than 500 people from the UK with mild-to-moderate intellectual disabilities. In their study, half of the participants identified “being able to connect with family and friends” as the best thing about the internet during the pandemic. Similarly, adults with intellectual disabilities indicated that being active in online events during the pandemic had positive impact on their well-being (Chadwick et al., 2023; Spassiani et al., 2022) In our study, the survey and interview questions about loneliness and well-being were limited, which restricted our possibilities to explore this topic further.

### Requirements and preconditions

Our study provided insight into the importance and presence of requirements for digital social contact within sheltered care facility homes. A stable internet connection was among the most important requirements, but was not provided by all care organisations. Lacking or poor internet connection has been identified as a barrier for digital participation of young people with intellectual disabilities in Sweden (Björquist & Tryggvason, 2023), as well as the use and implementation of eHealth within intellectual disability care in the Netherlands, both before (Frielink et al., 2021) and after the start of the pandemic (Oudshoorn et al., 2021).

Other important practical requirements were support with use and sufficient time for support, which were present in most cases. Support was not always present due to staff shortages, changes in staff schedules and lack of time. Another reason for the absence of support may be a lack of digital skills of staff members. Previous research suggested that barriers in supporting people with disabilities using digital social contact may be related to professional caregivers’ attitude, knowledge, and experience with technology (Björquist & Tryggvason, 2023; Hanzen et al., 2021; Nijs & Maes, 2021; Ramsten et al., 2017). Direct support staff in our study mentioned the availability of courses for digital social contact, but staff were not always able to take these courses due to their high workload. Organisational support for improving the digital skills of support staff may thus be important for their ability to support residents.

The extent to which residents needed support with using digital social contact depended on their cognitive and physical abilities and on their reading and writing skills. Overall, residents with severe or multiple disabilities needed more support than residents with mild-to-moderate disabilities. In our study, not all residents required support with digital social contact, mostly because those residents were able to solve technical problems on their own. The ability to solve technical issues independently may have a positive impact on their feelings of autonomy. However, the availability of support from staff may still be important for providing an autonomy supportive environment (Frielink et al., 2018).

### Strengths and limitations

A strength of the current study was the inclusion of the views and perceptions of residents, their relatives, volunteer visitors, direct support staff and care facility managers. However, the samples cannot be regarded as representative of the respective populations. People who responded to the questionnaires and interviews may have been more actively involved in using digital social contact than people who did not choose to participate (a limitation also noted by Araten-Bergman & Shpigelman, 2021). Nonetheless, some participants did not use digital social contact. Another strength of this study was the use of multiple methods. The interviews allowed us to explore experiences that would be difficult to capture in an online questionnaire. However, because the questionnaire was anonymous, it was not possible to follow up on questionnaire responses during the interviews. In addition, people with severe and profound intellectual disabilities were underrepresented in our study, because these participants were not able to fill in the questionnaire or participate in an interview on their own or with assistance. Similarly, the subsample of care facility managers was relatively small, with only 18 respondents to the questionnaire and four interviewees. This limited possibilities for investigating how digital social contact is implemented in care facility policies, which is a direction for future work.

### Directions for future studies

This was a cross-sectional study conducted during the COVID-19 pandemic. Longitudinal studies are needed to explore potential changes in the use of digital social contact by residents and their informal social networks, after the pandemic as compared to during the pandemic. Future research may also further explore differences in the use of and experiences with digital social contact between people with different kinds of disabilities. For instance, people with visual and intellectual disabilities or deafblindness may have different support needs than people without such conditions (see Dyzel et al., 2020). In addition, future work could further explore the links between digital social contact and well-being, including both the perspectives of people with disabilities and their family and friends. Lastly, more research should focus on the experiences of people with severe and profound intellectual disabilities, using methods such as observations and phenomenological analysis (Maes et al., 2021) and including significant others and proxies (Caton et al., 2023; Gjermestad et al., 2022).

### Conclusions and implications

This study adds to previous research about experiences with digital social contact by people with intellectual disabilities during the COVID-19 pandemic (e.g., Araten-Begman & Shpigelman, 2021; Caton et al., 2022; Chadwick et al., 2023; Embregts et al., 2020; Honingh et al., 2021). Our findings show that digital social contact, especially video calling, provides opportunities for both people with mild-to-moderate and severe or multiple disabilities to stay in touch with their informal social network. However, participants’ experiences were mixed. Benefits and disadvantages of using digital social contact may depend on residents’ abilities and support needs, as well as personal interests, opportunities for training, and the presence of preconditions, such as a stable internet connection and the availability of support staff. Overall, participants agreed that digital social contact cannot replace in-person contact, but many also noted the benefits of using it as complementary to in-person visits.

During the pandemic, many people started using digital means for social contact, including individuals with intellectual disabilities. Many residents and their relatives in the current study experienced digital social contact as meaningful and indicated that they would like to keep using it in addition to in-person contact, even after the pandemic. The current findings indicate the need for support professionals of care organisations to tailor to the needs of individual clients or residents, to increase their participation in the digital society. Specific implications for practice are outlined in our recent consensus statement (Douma et al., 2023), which is based on a systematic examination of the views and opinions of stakeholders regarding the use and facilitation of digital social contact within sheltered care facility homes. Lastly, the current findings emphasise the importance of including different perspectives. To improve the participation of people with disabilities in the digital world, it is important to include their voices, and those of other stakeholders, in the development of policy recommendations for digital social contact within care facility homes.
